# Fission Impossible: Stabilized miRNA-Based Analogs in Neurodegenerative Disease

**DOI:** 10.3389/fnins.2022.875957

**Published:** 2022-05-03

**Authors:** Walter J. Lukiw

**Affiliations:** ^1^LSU Neuroscience Center, Louisiana State University Health Science Center, New Orleans, LA, United States; ^2^Department of Ophthalmology, Louisiana State University Health Science Center, New Orleans, LA, United States; ^3^Department of Neurology, Louisiana State University Health Science Center, New Orleans, LA, United States

**Keywords:** Alzheimer's disease (AD), heterogeneity, miRNA-146a, microRNA, miRNA therapy, miRNA stabilization

## Overview

Small single-stranded non-coding ribonucleic acids (sncRNA) such as brain microRNA (miRNA) have biologically evolved for rapid post-transcriptional signaling to target messenger RNA (mRNA) and by doing so shape the transcriptome of the cell. Brain and central nervous system (CNS) miRNAs have typically short half-lives and information-carrying miRNA signals conveyed between the genome and the transcriptome are relatively rapid biological events. miRNAs are often mis-regulated in age-related neurological disorders such as Alzheimer's disease (AD). Chemically stabilized miRNAs and anti-miRNA oligonucleotides (antagomirs; AMOs) may be of therapeutic benefit in the treatment of neurological diseases exhibiting a specific down-regulation of brain-enriched miRNA or in diseases characterized by up-regulated miRNA signaling. The hippocampus is a major target of neuropathology in progressive age-related neurodegenerative disease and for the first time we present a table listing the most abundant miRNAs in the adult human hippocampal CA1 region. Also indicated on this list are 10 of the most significantly dysregulated miRNAs in this same anatomical region of AD brain. This opinion paper will address current research findings in this novel research area regarding the chemical stabilization of miRNA signaling as one innovative approach to the therapeutic management of neurological disease with specific reference to the human pro-inflammatory and innate-immune regulator miRNA-146a and AD-type neurodegeneration.

## The Complexity of Neurodegeneration

Human neurological disorders are generally complex, insidious and progressive neurodegenerative diseases and AD represents the most common form of senile dementia among our aging population (www.alz.org/media/documents/alzheimers-facts-and-figures.pdf; last accessed 30 March 2022). The molecular-genetic characterization and medical diagnosis of AD has turned out to be one of the most challenging in the history of clinical neurology. AD is most often associated with altered behavior, age-related memory deficits and progressive cognitive decline although less common clinical presentations have become increasingly recognized (https://alz-journals.onlinelibrary.wiley.com/doi/10.1002/alz.12328; last accessed 30 March 2022). The essential neuro-pathological features of AD have been known for almost ~120 years, and currently the presence and abundance of pathological lipoprotein-rich aggregates including amyloid-beta (Aβ) peptide-enriched “senile plaques” (SP) and the accumulation of abnormally hyper-phosphorylated tau (pTau) proteins into twisted neurofilament bundles known as neurofibrillary tangles (NFT) in the AD affected neocortex and hippocampus are still required for an accurate pathological diagnosis of AD at post-mortem examination of the brain (Alzheimer et al., 1995; DeTure and Dickson, 2019). Recent developments and advancements in RNA sequencing technologies and molecular imaging involving specialized positron emission tomography (PET)-based technologies and the use of multiple biofluid biomarkers have become increasingly useful for assisting in the early diagnosis, prognosis, prediction of time-to-symptom onset and drug treatment-monitoring of complex lipoproteinopathies including AD (Swarbrick et al., 2019; Shi et al., 2020; Wei et al., 2020; Zhao et al., 2020; Beach and Malek-Ahmadi, 2021; Ni and Nitsch, 2022; Ogonowski et al., 2022).

The significant heterogeneity in the presentation of AD is based in part on individual variation in genetics, genetic and familial history, the abundance and speciation of different SP and NFT isoforms in anatomical regions of the brain involved with cognition and memory, the Braak stage of the disease, the environment, diet and lifestyle, inter-current illness, multiple parameters associated with gender and aging and other factors associated with the intrinsic complexity of the disease itself (DeTure and Dickson, 2019; Habes et al., 2020). Since the first reported alterations of miRNA abundance, speciation and complexity in the affected regions of AD brain much research attention has been placed on: (i) the abundance, speciation, stability and lability of brain-enriched miRNAs; and (ii) how miRNA patterns are altered during the initiation and propagation of the neurodegenerative disease process as is observed both in affected AD tissues and in transgenic murine research models for AD (TgAD; Lukiw, 2007; Sobue, 2013; Bouter et al., 2020; Zhao et al., 2020; Lauretti et al., 2021; Pogue and Lukiw, 2021; Tasker et al., 2021).

## miRNAs in AD and Age-Related Neurodegeneration

microRNAs (miRNAs) are ~18-to-24 nucleotide (nt) sncRNAs that form a transient complex with other RNA-associated riboproteins such as Argonaute and bind to a 5–10 nt recognition “seed sequence” in the 3' untranslated region (3'-UTR) of their target mRNAs. In doing so miRNAs induce posttranscriptional repression of those mRNA targets in diverse eukaryotic lineages (Guo et al., 2010; Lukiw, 2012; Pogue et al., 2014; Bartel, 2018; Brennan and Henshall, 2020). The last 15 years of research has repeatedly confirmed that mammalian miRNAs predominantly act to decrease target mRNA levels to function in shaping the transcriptome of eukaryotic cells (Guo et al., 2010; Bartel, 2018; Ogonowski et al., 2022).

The total number of human miRNAs currently identified stands at about ~2,654 (miRBase Release 22.1; www.mirbase.org; https://lcsciences.com/services/microarray-services/mirna/; last accessed 30 March 2022) however expression array-based studies of miRNA temporal fluctuations, abundance and speciation shows both high dynamics and diversity that are significantly cell type-, developmental stage and tissue-specific (Li et al., 2013; Kozomara et al., 2019). In our 22-year study of extremely high quality samples of several hundred potential human brain neocortical and hippocampal short post-mortem interval (PMI) samples and biopsies for miRNA we are finding only about ~48 miRNAs relatively enriched in the human brain hippocampus, a limbic region of the brain highly susceptible to AD-tpe neuropathology (Table 1). An even smaller number of miRNAs appear to be altered in abundance in AD brain with about one third of all miRNAs upregulated and about two-thirds appearing as down-regulated miRNA species (Colangelo et al., 2002; Pogue et al., 2014; Bartel, 2018).

**Table 1:** Abundant miRNA species in the human brain hippocampus from a pool of 18 control brains.

hsa-let-7a**	hsa-miRNA-27b*	hsa-miRNA-128a*
hsa-let-7b*	hsa-miRNA-29a	hsa-miRNA-128b*
hsa-let-7c**	hsa-miRNA-30b*	hsa-miRNA-132*
hsa-let-7d**	hsa-miRNA-30c	hsa-miNA-143
hsa-let-7e*	hsa-miRNA-30d*	hsa-miRNA-145*
hsa-let-7f*	>hsa-miRNA-34a	hsa-miRNA-146a
hsa-let-7g**	hsa-miRNA-99a	>hsa-miRNA-155*
hsa-let-7i**	hsa-miRNA-99b	hsa-miRNA-181a*
hsa-miRNA-7*	hsa-miRNA-100	hsa-miRNA-181b
hsa-miRNA-9*	hsa-miRNA-103	hsa-miRNA-185
hsa-miRNA-16*	hsa-miRNA-107*	hsa-miRNA-191*
hsa-miRNA-23a	hsa-miRNA-124a*	hsa-miRNA-195
hsa-miRNA-23b	hsa-miRNA-125a	hsa-miRNA-221
hsa-miRNA-24	hsa-miRNA-125b**	hsa-miRNA-222*
hsa-miRNA-26a	hsa-miRNA-126	hsa-miRNA-320*
hsa-miRNA-26b	hsa-miRNA-127	hsa-miRNA-342*

*Most abundant miRNAs detected in the human brain hippocampus (hippocampal CA1 region); ranked by miRNA numerical designation; based on pooled data from N = 18 short post-mortem interval (PMI) controls; data derived from (Colangelo et al., 2002 and Jaber et al., 2017); this group included 9 males and 9 females, mean age 74 ± 7.2 years; all post-mortem interval (PMI; death to brain freezing interval at −81**°**C) were <3 h; each of these miRNAs yielded relatively high (>5,000) units of signal strength on an LC Sciences miRNA array Genechip (proprietary MRA-1001-miRNA microfluidic chip analytical platform; 2,650 small RNAs analyzed; LC Sciences Corporation, Houston, TX, USA; https://lcsciences.com/company/technology/technology-microarray/; last accessed 30 March 2022;) and ranked as the 48 most abundant miRNAs detected; except for miRNAs listed here, all other miRNAs are at a significantly lower abundance; note that there is a relatively high basal abundance of the let-7a to let-7g group of miRNAs; also note that AD-abundant miRNAs such as miRNA-146a and miRNA-155 exhibit high relatively variability in mean abundance in control brains, and are significantly induced as in AD brain, and especially as AD progresses; the 10 hippocampal-enriched miRNAs overlain in gray (and their mRNA targets) have been strongly implicated in AD neuropathology (Lukiw, 2007; Pogue and Lukiw, 2018; Mai et al., 2019; Swarbrick et al., 2019; Nunomura and Perry, 2020; Aslani et al., 2021; Grabowska-Pyrzewicz et al., 2021; Liang et al., 2021; Ogonowski et al., 2022); miRNAs with a single asterisk are especially abundant in control adult human hippocampus; miRNAs with two asterisks such as miRNA-125b and miRNA-7 are among the most abundant miRNAs in the human brain hippocampal CA1 region*.

The discovery and characterization of miRNAs, their temporal fluctuation in abundance and their biological actions in the developing, aging, and pathological human brain and CNS has opened a novel and fascinating vista into our appreciation of human brain epigenetics, the role of sncRNAs on homeostatic and pathogenic gene control and the potential role of sncRNA signals in modulating the genetic output of the human CNS (Guo et al., 2010; Bartel, 2018; Zhao et al., 2020; Pogue and Lukiw, 2021). Accordingly miRNAs, as the smallest known information-containing ribonucleic acids yet described, are associated with multiple cellular processes and the up-or down-regulation of miRNAs appears to be causative for numerous diseases including cancer, inflammation, cardiovascular disease and neurological disorders. Augmentation therapy using specific miRNAs has emerged as a promising approach through the use of stabilized synthetic miRNA or anti-miRNA mimics. Interestingly, in these diseases concatenated linear sncRNAs containing miRNA sequences may take the form of a circular RNA (circRNA) that have been found in multiple eukaryotic organisms including humans and may be especially abundant within the human CNS (Lukiw, 2013; Lauretti et al., 2021; Li et al., 2021; Su et al., 2022). Unlike linear miRNA, miRNA-containing circRNA may form a covalently closed loop lacking free 3' and 5' ends resulting in circRNAs being up to 5 times more stable than their linear counterparts with extended half-lives due to lack of exposed ends normally targeted by 3' or 5' exoribonucleases (Enuka et al., 2016; Lauretti et al., 2021). Interestingly one unusual circRNA has been described for miRNA-7 (ciRS-7) enriched in nervous tissues and contains about ~70 tandem anti-miRNA-7 sequences (Lukiw, 2013). ciRS-7 thereby acts as a kind of endogenous, competing, anti-complementary miRNA “sponge” to adsorb and hence quench miRNA-7 activities (Zhao et al., 2016; Lauretti et al., 2021). Stabilized circRNAs may have high therapeutic value in acting as long-lived “sponges” for the inactivation or down-regulation of overly expressed miRNAs in pathological conditions such as AD (Enuka et al., 2016; Mumtaz et al., 2020; Li et al., 2021; Su et al., 2022).

An attractive feature of stabilized miRNA as a pharmacotherapeutic agent is that miRNAs are involved in multiple highly interactive neuropathological signaling pathways. A single miRNA may target multiple mRNAs and hence multiple gene expression pathways, and multiple mRNAs may be targeted and down-regulated by multiple interdependent miRNA species (Bartel, 2018; Pogue and Lukiw, 2018; Nguyen et al., 2021). Interestingly, as few as 10 miRNAs or clustered miRNA families altered in abundance in AD hippocampus including miRNA-7, miRNA-9, miRNA-23a, miRNA-29, miRNA-30b, miRNA-34a, miRNA-107, miRNA-125b, miRNA-146a, miRNA-155 can explain most of the major features of AD neuropathology involving highly interactive molecular networks involved in amyloid and neurofibrillary homeostasis, altered NF-kB and innate-immune signaling, tau pathology, deficits in phagocytosis, catabolism, neurotrophism, synaptogenesis, inflammation and amyloido-genesis (Table 1; Lukiw, 2007; Millan, 2017; Pogue and Lukiw, 2018; Brennan and Henshall, 2020; Wei et al., 2020; Tasker et al., 2021; Walgrave et al., 2021).

## The Natural Decomposition of microRNA

Neurobiological pathways involved in the decay of mature brain-enriched miRNAs are less well-understood than those which mediate the biogenesis of these sncRNAs. Similar to mRNAs; (i) of all dinucleotide combinations, the percent AU or UA dinucleotide content (in either the 5′-3′ or 3′-5′ orientation) of miRNAs has shown the strongest observed correlation with the observed miRNA half-life (*r*^**2**^ = 0.95); and (ii) AU dinucleotide content of brain miRNAs is strongly correlated with intrinsic miRNA instability yielding miRNAs with half-lives (T_**1/2**_) in the range of 1.0–3.5 h (Sethi and Lukiw, 2009; Pogue et al., 2014; Bartel, 2018). Cellular miRNAs have been reported to be degraded in human cells by multiple endo- and exo-ribonuclease activities. These include an evolutionarily conserved endonuclease Tudor-SN (TSN) that contains five staphylococcal/ micrococcal nuclease-like SN-domains and a structurally conserved methylation-state specific Tudor domain which degrades both protein free and Argonaute-loaded miRNAs via endonucleolytic cleavage at the dinucleotides CA, AU, or UA located more than 5 nt away from the 3' or 5' miRNA termini (Elbarbary et al., 2017; Li et al., 2018). Other TSN-type staphylococcal endoribonucleases and RNA-binding proteins (RNABPs) also appear to be involved in miRNA stability (Li et al., 2018; Kinoshita et al., 2021). In the brain and CNS miRNA half-lives may be significantly extended by miRNA binding to ribonucleoproteins (RNPs), by primary structure folding of the miRNA ribonucleotide backbone itself into protected structures, by compartmentalization of miRNAs into exosomes (EXs), ectosomes (ECs) or extracellular microvesicles (EMVs) or by any combination of these (Lukiw and Pogue, 2020; Kinoshita et al., 2021; Meldolesi, 2021). Interestingly, the molecular cargoes of EXs, ECs and EMVs that include various mixtures of peptides, proteins, lipids, proteolipids, cytokines, chemokines, carbohydrates, multiple species of miRNAs, mRNAs, RNPs and other components, including end-stage neurotoxic and pathogenic metabolic products, such as Aβ42 peptides may also associate with miRNAs and enhance their stability as part of a highly complex and dynamic system of intercellular communication within the brain (Elbarbary et al., 2017; Lukiw and Pogue, 2020; Kinoshita et al., 2021).

## The Modulation of microRNA Stability

The use of synthetic, chemically modified and stabilized miRNAs or anti-miRNA oligonucleotides (antagomirs) represents a novel direction and innovative application in the utilization of sncRNAs in the therapeutic management of neurodegenerative disease. For at least ~25 years mRNA-sized synthetic oligonucleotides have been in use employing different mechanisms of action from steric hindrance to diminished or enhanced degradation by endogenous RNase H- or L-type exo- and endo-nucleases naturally abundant in the cellular environment (Lennox and Behlke, 2011; Evers et al., 2015; Grabowska-Pyrzewicz et al., 2021). Optimized biochemical modification strategies originally designed for large mRNAs (~2,000–5,000 nt) have aided in the strategic design and current use of miRNA-based therapeutics (Lima et al., 2018; Liang and Wang, 2021; Nguyen et al., 2021). Chemical modification of synthetic sncRNA oligonucleotides can instill nuclease resistance, increase binding affinity to mRNA 3'-UTR ‘seed sequence' regions and complexation with lipophilic molecules such as cholesterol or other lipids can aid in the cellular uptake and/or targeting by nucleases (Simonson and Das, 2015; Nguyen et al., 2021; Walgrave et al., 2021). The stabilization of miRNAs via small-molecule inhibition of RNase L and other ribonucleases can further increase the longevity and efficiency of miRNA-mediated replacement therapy (Lima et al., 2018; Nogimori et al., 2019; Nguyen et al., 2021; Walgrave et al., 2021).

Multiple miRNA chemically-based stabilization strategies can involve: (i) the modification of miRNA phosphates (such as the use of phosphorothioates); (ii) ribose sugar ring modifications including the use of locked nucleic acids (LNAs; novel bicyclic nucleic acids that tether the 2′-*O* to the 4′-*C* via a methylene bridge effectively locking the structure into a 3′-endo sugar conformation); (iii) the use of high affinity nuclease resistant 2′-*O*-methyl RNA chemistries (2'*O-*Me); (iv) the application of nuclease resistant 2'-fluoro (2'-F) RNA strategies that stabilize 2'-F-containing miRNAs; (v) 2′-*O*-methyoxyethyl (2'-M*O*E) modifications; and (vi) any combination of these that convey ribonuclease resistance and/or increases miRNA-mRNA “seed sequence” binding (Lennox and Behlke, 2011; De Clercq, 2013; Lima et al., 2018; Silva et al., 2020). Non-ribose backbone modifications including phosphorodiamidate morpholino oligonucleotides (PMOs) and peptide nucleic acids (PNAs) have shown promise as steric blocking antisense agents, and when conjugated with cell-penetrating peptides or molecules that facilitate delivery into mammalian cell systems via intravenous or intraperitoneal injection allow the rapid penetration of a wide range of cellular membranes and biophysical barriers including the blood-brain barrier (Lima et al., 2018; Nguyen et al., 2021; Walgrave et al., 2021). The use and details of these novel stability-augmenting chemistries for sncRNAs and miRNAs have been extensively discussed and reviewed over the last decade in a series of comprehensive studies and detailed reports some of which are listed here (Lennox and Behlke, 2011; De Clercq, 2013; Baigude and Rana, 2014; Evers et al., 2015; Elbarbary et al., 2017; Lima et al., 2018; Mai et al., 2019; Silva et al., 2020; Grabowska-Pyrzewicz et al., 2021; Groaz and De Jonghe, 2021; Seley-Radtke et al., 2021).

## Experimental and Therapeutic Applications of Stabilized microRNA

miRNA profiles in the brains of neurodegenerative disease patients are widely documented to be significantly altered compared to healthy age-matched controls, often in a disease stage- and/or anatomically-specific manner and correlated with the neuropathological anatomy of the disease. In AD, how these specific alterations in miRNA abundance, speciation and complexity impact disease initiation, propagation and severity and whether they are the result, cause or effect, along the trajectory of this heterogeneous and complex disease in most cases remains unclear (Colangelo et al., 2002; Lukiw, 2007; Herrera-Espejo et al., 2019; Kinoshita et al., 2021; Li and Cai, 2021; Liang et al., 2021; Pogue and Lukiw, 2021). Specific drug targeting issues and complications can be overcome by extensive experimentation involving testing with differentially stabilized miRNAs, antagomirs and related anti-ribonuclease strategies, the updating and constant refinement of miRNA-based therapeutic approaches and by novel miRNA and/or antagomir delivery protocols using TgAD models as a guide (Kinoshita et al., 2021; Liang and Wang, 2021; Liang et al., 2021; Nguyen et al., 2021; Zhang et al., 2021). The route of administration into the brain of chemically modified and/or stabilized miRNA species, such as via intranasal, intrathecal or direct intracerebroventricular infusion often enables the functional delivery of stabilized oligonucleotides in the absence of any delivery vehicle both in murine models and in humans (Mai et al., 2019; Nogimori et al., 2019; Silva et al., 2020; Maimon et al., 2021; Nguyen et al., 2021).

In one of the earliest studies a stabilized antagomir of the pro-inflammatory and innate-immune regulator miRNA-146a (known to be significantly up-regulated in AD hippocampus), after intra-hippocampal delivery into an amyloid-over-expressing 5xFAD TgAD murine model (containing five familial AD-relevant mutations) was shown to repress tau hyperphosphorylation and in part restore cognition and memory function (Wang et al., 2016; Zhang et al., 2021). In a related study, upregulated miRNA-146a was targeted using a mouse-specific miRNA-146a LNA tethered to a cholesterol carrier and using a non-invasive nasal administration it was demonstrated that this antagomir (M146AG) rescued cognitive impairment and restored memory in an APP/PS1 transgenic murine model for AD and alleviated the overall neuropathological progression. This included a down-regulation of neuro-inflammatory signaling, reduced glial activation and a reduction of Aβ peptide deposition and tau phosphorylation in hippocampal regions of the brain (Mai et al., 2019). In more recent study a 2'-methoxy-modified and stabilized miRNA-146a antagomir after direct intra-hippocampal delivery, functioned via competitive binding to inhibit miRNA-146a signaling in an APP/PS1 TgAD murine model to oppose the pathological process of cognitive failure mainly through the augmentation of neuro-inflammation-related pathways (Liang et al., 2021). In a related approach and targeting the polypyrimidine tract binding protein 1 (PTBP1) using a stabilized ~20 nt antisense oligonucleotide containing 2′-methoxyethyl (MOE)-modified nucleotides, and delivered by a single injection into the CSF, it was shown that the new neurons generated functionally integrated into endogenous circuits and modified mouse behavior, opening up the prospect that production of new neurons could replace those lost to the neurodegenerative disease process associated with both advanced aging and AD (Maimon et al., 2021). Presumably, these new neurons produced in the aging murine brain contained a complete repertoire of fully functional miRNAs and miRNA-mediated signaling pathways conducive to the restoration of homeostatic brain regulatory function. One major caveat to these animal studies is that very often stabilized miRNA-based analogs used in murine models of human neurodegenerative disease have shown great promise while their clinical application to the human condition has often been fraught with difficulties including off-target effects. These ongoing concerns have been thoughtfully addressed and reviewed in several recent papers (Reddy et al., 2020; Lauretti et al., 2021; Walgrave et al., 2021).

## Discussion and Summary

The utilization of chemically stabilized miRNAs, antagomirs and/or anti-ribonuclease strategies in the clinical management of neurodegenerative disease for therapeutic benefit is a relatively recent one (Wei et al., 2020; Kinoshita et al., 2021; Lauretti et al., 2021; Nguyen et al., 2021). One confounding feature of targeted miRNA- antagomir-based therapies are the spatial and temporal fluctuations in abundance and speciation of specific miRNAs in any one cell type and the cellular specificity of these same sncRNAs. For example the human hippocampal-enriched miRNA-107 appears to decrease as AD progresses while miRNA-146a increases both in the hippocampus in both AD and in murine TgAD models (Table 1; Lukiw, 2012; Millan, 2017; Mai et al., 2019). Certain hippocampal-enriched miRNAs such as members of the miRNA-29 microRNA family (miRNA-29a, miRNA-29b and miRNA-29c) exhibit different expression patterns in the hippocampus, being up-regulated in the early stages of AD in transgenic murine models while being significantly downregulated in the other regions of the brain cortex as AD-type neurodegeneration progresses (Zong et al., 2015; Wei et al., 2020). Different temporal delivery approaches using appropriate miRNA, antagomir and/or anti-ribonuclease treatments should be useful in the more effective application of miRNA-based therapeutic strategies in these complex situations where specific pathological miRNA levels are in fluctuation during the course of the disease (Lima et al., 2018; Wei et al., 2020; Nguyen et al., 2021).

Several important areas enjoying unanticipated, unexpected and perhaps serendipitous success are: (i) that exogenously applied stabilized miRNA mimics and/or antagomirs may be shuttled to cellular compartments which are the cell's natural location already enriched in these same miRNAs; (ii) this trafficking may in part be mediated by EXs, ECs and EMVs utilized by all cell lineages of human neural cells and part of a highly complex and dynamic system of intercellular communication within the brain and CNS; and (iii) that certain critical miRNAs such as miRNA-146a operate at multiple neurobiological levels to regulate multiple mRNAs responsible for several key aspects of AD neuropathology (Elbarbary et al., 2017; Jaber et al., 2017; Lima et al., 2018; Lukiw and Pogue, 2020; Kinoshita et al., 2021; Meldolesi, 2021; Nguyen et al., 2021).

Key challenges for miRNA-based therapeutic optimization including direct miRNA modification, antagomir and circRNA-based design strategies and their application and the selection and targeting of appropriate miRNA-mediated, anatomically- and disease-relevant neuropathological signaling pathways are an urgent and continuing goal (Lennox and Behlke, 2011; Baigude and Rana, 2014; Evers et al., 2015; Zhao et al., 2016; Silva et al., 2020; Nguyen et al., 2021). The expanding incidence of human neurodegenerative disorders such as AD and the general low rates of success of neurological drug trials are natural drivers for the implementation of these types of miRNA-related stabilization and delivery systems, especially since no effective treatment options for AD are currently available (Lauretti et al., 2021; Nguyen et al., 2021; Walgrave et al., 2021). One primary reason for these disappointing results may be that clinical trials enroll patients with neurodegenerative disorders such as AD at advanced stages when the disease has already taken hold. Although many drugs and agents are effective in TgAD murine models, have been tested pre-clinically and show promising results, in human clinical trials they are ineffective in slowing AD progression (Hu et al., 2016; Reddy et al., 2020; Kinoshita et al., 2021; Walgrave et al., 2021). Off-target side effects are currently another major hurdle for miRNA-based therapeutics to overcome in order to transfer knowledge gained from cell culture and transgenic animal modeling to the AD clinic.

Lastly, for CNS applications, antibody-, peptide- cholesterol- and/or other lipid or proteolipid-oligonucleotide-miRNA conjugates offer a promising strategy for the efficient delivery of sncRNA-based therapeutics across the blood brain barrier to ultimately access specific brain cell types (Lima et al., 2018; Nguyen et al., 2021; Walgrave et al., 2021). The exploitation of naturally occurring intra- and inter-cellular vesicle-mediated delivery and trafficking pathways that transport miRNA or other small molecules including miRNA-selective nucleases should be particularly useful in the application and successful implementation of stabilized miRNA-based therapeutics across a broad spectrum of human neurodegenerative disease.

## Author Contributions

WL collected and analyzed all data and researched and wrote the article.

## Funding

Research involving sncRNA and miRNA signaling, the innate-immune response, amyloidogenesis and neuro-inflammation in AD, and prion disease and anti-miRNA therapeutic strategies was supported through the Louisiana Biotechnology Research Network (LBRN), an unrestricted grant to the LSU Eye Center from Research to Prevent Blindness (RPB), The Brown Foundation, Joe and Dorothy Dorsett Innovation in Science Healthy Aging Award, and National Institutes of Health (NIH) NIA Grants AG18031 and AG038834.

## Conflict of Interest

The author declares that the research was conducted in the absence of any commercial or financial relationships that could be construed as a potential conflict of interest.

## Publisher's Note

All claims expressed in this article are solely those of the authors and do not necessarily represent those of their affiliated organizations, or those of the publisher, the editors and the reviewers. Any product that may be evaluated in this article, or claim that may be made by its manufacturer, is not guaranteed or endorsed by the publisher.
